# Supplementary material for: The Systems Biology Research Tool: evolvable open-source software
Source: BMC Syst Biol. 2008 Jun 29;2:55. doi: 10.1186/1752-0509-2-55 (PMC2446383; doi:10.1186/1752-0509-2-55)
Supplement: Additional file 1 — SBRT Archive. An archive of the current version of the Systems Biology Research Tool. [file 1752-0509-2-55-S1.zip › sbrt-1.4.0/doc/developers_guide/api/sbrt/kernel/math/algebra/HashLinearSystemSolution.html]

HashLinearSystemSolution


|  |  |  |
| --- | --- | --- |
| **Overview** \| **Package** \| **Class** \| **Use** \| **Tree** \| **Deprecated** \| **Index** \| **Help** | |  |
| **PREV CLASS**   **NEXT CLASS** | **FRAMES**    **NO FRAMES**     **All Classes** |  |
| SUMMARY: NESTED \| FIELD \| CONSTR \| METHOD | DETAIL: FIELD \| CONSTR \| METHOD |  |


---


## sbrt.kernel.math.algebra Class HashLinearSystemSolution<V>

```
java.lang.Object
  sbrt.kernel.math.algebra.HashLinearSystemSolution<V>
```

**Type Parameters:**: `V` - the variable type.

**All Implemented Interfaces:**: LinearSystemSolution<V>

---

``` public class HashLinearSystemSolution<V> extends java.lang.Object implements LinearSystemSolution<V> ```

This class is a concrete implemenation of `LinearSystemSolution`. The
hash codes of the provided variables are used extenstively in this
implementation to provide constant-time lookup for internally stored
information.

**Author:**
:   This class was written and documented by Jeremiah Wright while in the
    Wagner lab.

---

**Constructor Summary**

`HashLinearSystemSolution(java.util.Map<? extends V,? extends LinearComb<? extends V>> solutions)`
  
          Constructs a new hash code-based linear system solution.


| **Method Summary** | |
| --- | --- |
| `int` | `fixedVariables()`             Returns the number of fixed variables in this solution. |
| `int` | `freeVariables()`             Returns the number of free variables in this solution. |
| `java.util.Set<V>` | `getFreeVariables()`             Returns the set of free variables contained in this solution. |
| `LinearComb<V>` | `getSolution(java.lang.Object variable)`             Returns the linear combination of free variables to which the provided variable is equal. |
| `java.util.Map<V,java.lang.Double>` | `getSolutionVector(java.util.Map<?,java.lang.Double> freeVariableValues)`             Computes and returns a solution vector by plugging in the provided values for the free variables in this solution. |
| `java.util.Set<V>` | `getVariables()`             Returns the set of variables contained in this solution. |
| `boolean` | `isSolutionVector(java.util.Map<?,java.lang.Double> point, double tolerance)`             Indicates if the provided solution vector is consistent with this solution. |
| `boolean` | `isVariable(java.lang.Object obj)`             Indicates if the provided object is a variable in this solution. |
| `static <V> LinearSystemSolution<V>` | `simplify(LinearSystemSolution<V> solution, java.util.Map<? extends V,Interval> variableBounds, double tolerance)`             Returns a simplified version of the provided linear system solution given the variable bounds. |
| `LinearSystemSolution<V>` | `simplify(java.util.Map<?,java.lang.Double> variableValues, double tolerance)`             Computes and returns a simplified solution by plugging in the provided values. |
| `java.util.Map<V,LinearComb<V>>` | `toMap()`             Returns this solution as a map. |
| `int` | `variables()`             Returns the total number of variables in this solution. |

**Methods inherited from class java.lang.Object**

`clone, equals, finalize, getClass, hashCode, notify, notifyAll, toString, wait, wait, wait`

**Constructor Detail**

### HashLinearSystemSolution

```
public HashLinearSystemSolution(java.util.Map<? extends V,? extends LinearComb<? extends V>> solutions)
```

:   Constructs a new hash code-based linear system solution.

    **Parameters:**: `solutions` - variables mapped to the linear combination of free variables to which they equal. The set of variables in the provided linear combinations must be a subset of the keys in the provided map. **Throws:**: `MissingVariableException` - if at least one free variable does not appear as a key in the provided map.


**Method Detail**

### simplify

```
public static <V> LinearSystemSolution<V> simplify(LinearSystemSolution<V> solution,
                                                   java.util.Map<? extends V,Interval> variableBounds,
                                                   double tolerance)
```

:   Returns a simplified version of the provided linear system solution
    given the variable bounds.

    :   **Type Parameters:**: `V` - the variable type. **Parameters:**: `solution` - a solution to a system of linear equations.: `variableBounds` - the bounds on each variable in that solution.: `tolerance` - the tolerance used to simplify the provided linear system solution. See `LinearSystemSolution.simplify(Map, double)`. **Returns:**: a simplified version of the provided linear system solution given the variable bounds.

---


### variables

```
public int variables()
```

:   Returns the total number of variables in this solution. This equals the
    sum of free and fixed variables.

    :   **Specified by:**: `variables` in interface `LinearSystemSolution<V>`
    :   **Returns:**: the total number of variables in this solution. **See Also:**: `freeVariables()`, `fixedVariables()`

---


### freeVariables

```
public int freeVariables()
```

:   Returns the number of free variables in this solution.

    :   **Specified by:**: `freeVariables` in interface `LinearSystemSolution<V>`
    :   **Returns:**: the number of free variables in this solution.

---


### fixedVariables

```
public int fixedVariables()
```

:   Returns the number of fixed variables in this solution.

    :   **Specified by:**: `fixedVariables` in interface `LinearSystemSolution<V>`
    :   **Returns:**: the number of fixed variables in this solution.

---


### getVariables

```
public java.util.Set<V> getVariables()
```

:   Returns the set of variables contained in this solution.

    :   **Specified by:**: `getVariables` in interface `LinearSystemSolution<V>`
    :   **Returns:**: the set of variables contained in this solution.

---


### getFreeVariables

```
public java.util.Set<V> getFreeVariables()
```

:   Returns the set of free variables contained in this solution.

    :   **Specified by:**: `getFreeVariables` in interface `LinearSystemSolution<V>`
    :   **Returns:**: the set of free variables contained in this solution.

---


### getSolution

```
public LinearComb<V> getSolution(java.lang.Object variable)
```

:   Returns the linear combination of free variables to which the provided
    variable is equal.

    :   **Specified by:**: `getSolution` in interface `LinearSystemSolution<V>`
    :   **Parameters:**: `variable` - a variable from this solution. **Returns:**: the linear combination of free variables to which the provided variable is equal.

---


### toMap

```
public java.util.Map<V,LinearComb<V>> toMap()
```

:   Returns this solution as a map.

    :   **Specified by:**: `toMap` in interface `LinearSystemSolution<V>`
    :   **Returns:**: the variables of the original system of equations mapped to the linear combination of free variables to which they equal.

---


### getSolutionVector

```
public java.util.Map<V,java.lang.Double> getSolutionVector(java.util.Map<?,java.lang.Double> freeVariableValues)
```

:   Computes and returns a solution vector by plugging in the provided values
    for the free variables in this solution.

    :   **Specified by:**: `getSolutionVector` in interface `LinearSystemSolution<V>`
    :   **Parameters:**: `freeVariableValues` - free variables mapped to their corresponding double precision value. Every key in the map must be a free variable in this solution and all free variables must be present. **Returns:**: the solution vector resulting from the provided free variable values.

---


### simplify

```
public LinearSystemSolution<V> simplify(java.util.Map<?,java.lang.Double> variableValues,
                                        double tolerance)
```

:   Computes and returns a simplified solution by plugging in the provided
    values. Values can be provided for both free and fixed variables.

    :   **Specified by:**: `simplify` in interface `LinearSystemSolution<V>`
    :   **Parameters:**: `variableValues` - variables mapped to their corresponding double precision value. Every key in the map must be a variable in this solution.: `tolerance` - the amount by which a provided or computed value is allowed to differ from any previously existing value. This should be a small positive number, like 1E-9 for example. **Returns:**: the solution resulting from plugging in the provided values.

---


### isVariable

```
public boolean isVariable(java.lang.Object obj)
```

:   Indicates if the provided object is a variable in this solution.

    :   **Specified by:**: `isVariable` in interface `LinearSystemSolution<V>`
    :   **Parameters:**: `obj` - the potential variable. **Returns:**: `true` if the provided object is a variable in this solution; `false` otherwise.

---


### isSolutionVector

```
public boolean isSolutionVector(java.util.Map<?,java.lang.Double> point,
                                double tolerance)
```

:   Indicates if the provided solution vector is consistent with this
    solution.

    :   **Specified by:**: `isSolutionVector` in interface `LinearSystemSolution<V>`
    :   **Parameters:**: `point` - the variables of this solution mapped to their values. Every key in the map must be a variable in this solution and all variables must be present.: `tolerance` - the amount by which a provided value is allowed to differ from any computed or previously existing value. This should be a small positive number, like 1E-9 for example. **Returns:**: `true` if the provided vector is consistent with this solution; `false` otherwise.


---


|  |  |  |
| --- | --- | --- |
| **Overview** \| **Package** \| **Class** \| **Use** \| **Tree** \| **Deprecated** \| **Index** \| **Help** | |  |
| **PREV CLASS**   **NEXT CLASS** | **FRAMES**    **NO FRAMES**     **All Classes** |  |
| SUMMARY: NESTED \| FIELD \| CONSTR \| METHOD | DETAIL: FIELD \| CONSTR \| METHOD |  |


---
